# The Proximate Cause of Insanity

**Published:** 1853-10-01

**Authors:** 


### THE PROXIMATE CAUSE OF INSANITY.

In the thirteenth and fourteenth numbers of *The Psychological Journal* we entered very fully into the consideration of a "new theory of insanity," propounded by Dr. Henry Monro; but while we admired the philosophical spirit in which it was conceived and enunciated, and were especially delighted with the graceful style of the writer, we were compelled to dissent from his conclusions, and even to withhold from him the praise of originality for the theory itself. In his views of treatment —in his incidental reflections upon various mental states, and in most of his moral speculations, we cordially concurred, and felt on closing his elegant essay that we had been in pleasant communion with an honest truth-seeker, who fully believed in the originality of his doctrine, and was influenced by pure motives in publishing them to the world. The object of the work* before us, is still further to elucidate and substantiate the *same theory*, and to claim for the author of the volume, the merit of having been the first to bring before the world the views propounded in Dr. Henry Monro's book. We confess that we are not aware that the theory was distinctly announced by the present author in his former publications—although enough was stated to establish inferentially a corresponding idea in the mind of the writer, but in a manner, as in no way to detract from the claims, such as they are, of the preceding theorist. We have heretofore shown that the *asthenic* character of *some forms* of insanity had been fully recognised by Hill, Crichton, and others, and that Dr. Henry Monro differed from these authors (as indeed does Dr. Davey) only by limiting the disease to one especial cause, such as deficiency of nervous tone, and in doing so, has, we conceive, rendered "the theory and opinion unsound, because not in harmony with *all* the facts of the case."

There is no difference between the theory of Monro and Davey, and we have therefore nothing to add to, or subtract from, our former observations, respecting its merits or truth.

In justice, however, to our own position, we wish to make a few remarks on an incidental statement in Dr. Davey's essay. The statement is as follows:—"Throughout the country, *psychological journals* exist and enjoy a very respectable position, but are nevertheless *without the first principles of a sound* and inductive psychology. The name of Gall can hardly be found on their closely-printed pages," page 25. The same statement, or rather opinion, of what only is "*sound* psychology"

* On the Nature and Proximate Cause of Insanity. By Dr. Davey. Churchill. London.

is expressed in equally strong language in Dr. Davey's former work "On Mental Pathology:"—"No person, *unless he be a phrenologist*, that is, unless he is well acquainted with the functions of the brain in a state of health (! !) can *possibly* be a good judge of the indications of an unsound mind," page 91. Dr. Davey writes in the plural number respecting the "psychological journals" of this country, but in our own behalf, we protest against the truth of his statements. The "name of Gall" is as familiar as a "household word" to the readers of this journal, and we have endeavoured to put forth whatever is valuable in his theory, while his anatomical researches have ever received our warmest praise. We are not fanatical followers of any name, however great, and claim it as our right, and imperative duty, to submit *all* theories to a rigid and scrutinizing analysis, and compare them most carefully with well-observed and established facts. If the conventional meaning of the term "phrenology" be departed from, and the word be made to embrace the large signification modestly assigned to it by Dr. Davey, and which, we confess, its derivation (φρην-λογος) would imply, then have we no cause to complain of his argument; but if the term embrace only the theory of Gall (craniology), then, the sentence which we have quoted, short as it is, embraces two arguments wholly unconnected with each other. It intimates that "phrenology" and "the functions of the brain" are convertible terms—a dogmatism against which we protest, as we do to the corollary, that no person can *possibly* be "a good judge of the indications of an unsound mind unless he be well acquainted with the functions of the brain in a state of health." The functions of *the brain* are at this moment unknown. The functions of some parts of the structure are alone understood. A great portion of this important organ has still its function, or functions, to be discovered. Does the present state of cerebral "*pathology*" support the belief that phrenology reveals the functions of the *entire* brain? The exterior of the brain is mapped out, by Combe, into *sixty-six organs*—but what of the huge masses of "grey matter" in the interior, at the base, and in the inner lateral portions of the cerebral hemispheres?

But apart from this anatomical defect, has any anatomist of repute published a case in which the autopsy revealed cerebral lesion limited to one of the above topographical regions, and this region according with the impaired or diseased mental manifestations during life? For example, has a defect in the organ of comparison (the mental attribute which, according to Dr. Conolly, exerts so great an influence in the production or control of insanity) been discovered by the scalpel in a sufficient number of cases, to set aside the possibility of coincidence? Mr. Lawrance, whose mental bias, early studies, and published writings, lead him to adopt the opinions of the Sensational School of Philosophy, has published no such case, although the brains of all the lunatics who die at Bethlem Hospital are examined by him. The results of several hundred examinations have been published, but, in these researches, we look in vain for any circumscribed or phrenological lesion, or for any details which will uphold the hypothesis so boldly asserted in the above paragraphs. If we extend our investigations still further, and examine the numerous facts recorded by Esquirol, we not only find no support, but meet with a positive denial of all coincidence between the morbid lesions of the cerebrum and the theory of phrenology. At Ivry, Esquirol had a very large collection of skulls, and casts from the heads of lunatics, and he positively assured the late Dr. Prichard, that during his long career of observation at the Salpêtrière, and at Charenton, he had not met anything which supported the above hypothesis; and that, in short, his whole experience was "entirely adverse to the doctrines of the phrenologists." "This observation," says Dr. Prichard, "was made in the presence of Monsieur Mativiê, and received his assent and confirmation." The experience of Foville, the late able and observant superintendent of the asylum of St. Yon, accords with the above; and the opinions of the resident medical officers at Hanwell, in 1850, were to the same effect. With a strange inconsistency, the author states, in his "Mental Pathology," that Dr. Prichard was the first to detect and to describe the form of disease called moral insanity; and yet, "no person, unless he be a phrenologist, can *possibly* be a good judge of the indications of unsound mind." The illustrious dead are thus libelled as blunderers. Esquirol, Haslam, Burrows, Pinel, and Prichard, repudiated or disbelieved in the hypothesis, and no one until now has had the boldness to declare that they could not "*possibly*" be good judges of the indications of unsound mind. Jacobi, the talented and experienced physician of the Seigburg Asylum, belongs to the same list of noble dissentients, and we may, therefore, as journalists, be well satisfied to be rebuked with them, as being destitute of "the first principles of a sound psychology." We do not, however, deny that the phrenological theory is ingenious, and with certain modifications, deserves the attention of psychologists; but we hold that Dr. Davey has outstepped the limits of strict induction, and entered the arena of sheer dogmatism, by upbraiding us, as destitute "of the first principles of a sound psychology," solely because we cannot follow him in his enthusiastic admiration of the writings and labours of Dr. Gall.

But to return briefly to Dr. Davey's theory of insanity, we may observe that "morbid sensibility," or irritation, are convenient phrases to use, and most difficult conditions to refute—inasmuch as when in existence they admit of no appeal to the senses, no demonstration, and are regarded by their advocates, as the antecedents of whatever physical or pathological changes may be detected in the cerebral or other structures after death. As we stated in reviewing the elegant essay of Dr. Monro upon the same subject—a low tone of vitality and "irritation" or morbid "sensibility" consequent upon this, may, as a truism, be accepted as the condition of all disease, but it remains to be proved, that it is especially the "*fons et origo mali*" in all cases of mental derangement. The ardent mind of Dr. Davey has impulsively embraced this theory, because at Hanwell and elsewhere he saw many patients who recovered rapidly from their respective maladies by a judicious course of tonic treatment; but a careful perusal of the cases recited from his practice at Hanwell, Colombo, and Colney Hatch, will prove to the unbiassed mind that the irritation, or "morbid sensibility" referred to, was in some cases dependent upon derangement of the digestive organs, and the others, upon an impoverished condition of the blood. It gives us much pleasure, however, to state, that the method of treatment inculcated in this little essay is most judicious, and that the general statements which pervade the work are such as do honour to the head and heart of the writer.

